# Molecular Cloning, Recombinant Expression and Antifungal Activity of BnCPI, a Cystatin in Ramie (*Boehmeria nivea* L.)

**DOI:** 10.3390/genes8100265

**Published:** 2017-10-11

**Authors:** Yongting Yu, Gang Zhang, Zhimin Li, Yi Cheng, Chunsheng Gao, Liangbin Zeng, Jia Chen, Li Yan, Xiangping Sun, Litao Guo, Zhun Yan

**Affiliations:** 1 Institute of Bast Fiber Crops and Center for Southern Economic Crops, Chinese Academy of Agricultural Science, Changsha 410205, China; Lizhimin@caas.cn (Z.L.); Chengyi@caas.cn (Y.C.); Csgao06@163.com (C.G.); Zengliangbin@caas.cn (L.Z.); Chenjia01@caas.cn (J.C.); Yanli214@126.com (L.Y.); Sunxp66@163.com (X.S.); guolitao@caas.cn (L.G.); 2 College of Pharmacy and Shaanxi Provincial Key Laboratory for Chinese Medicine Basis & New Drugs Research, Shaanxi University of Chinese Medicine, Xi’an 712406, China; jay_gumling2003@aliyun.com

**Keywords:** *Boehmeria nivea* L., BnCPI, cysteine protease inhibitors, intron, functional expression

## Abstract

Phytocystatins play multiple roles in plant growth, development and resistance to pests and other environmental stresses. A ramie (*Boehmeria nivea* L.) phytocystatin gene, designated as *BnCPI*, was isolated from a ramie cDNA library and its full-length cDNA was obtained by rapid amplification of cDNA ends (RACE). The full-length cDNA sequence (691 bp) consisted of a 303 bp open reading frame (ORF) encoding a protein of 100 amino acids with deduced molecular mass of 11.06 kDa and a theoretical isoelectric point (pI) of 6.0. The alignment of genome DNA (accession No. MF153097) and cDNA sequences of *BnCPI* showed that an intron (~104 bp) exists in the coding region. The BnCPI protein contains most of the highly conserved blocks including Gly^5^-Gly^6^ at the N-terminal, the reactive site motif QxVxG (Q^49^V^50^V^51^S^52^G^53^), the L^79^-W^80^ block and the [LVI]-[AGT]-[RKE]-[FY]-[AS]-[VI]-x-[EDQV]-[HYFQ]-N (L^22^G^23^R^24^ F^25^A^26^V^27^ D^28^D^29^H^30^ N^31^) block that is common among plant cystatins. BLAST analysis indicated that BnCPI is similar to cystatins from *Glycine max* (77%), *Glycine soja* (76%), *Hevea brasiliensis* (75%) and *Ricinus communis* (75%). The *BnCPI* was subcloned into expression vector pSmart-I and then overexpressed in *Escherichia coli* BL21 (DE3) as a His-tagged recombinant protein. The purified reBnCPI has a molecular mass of 11.4 kDa determined by sodium dodecyl sulfate polyacrylamide gel electrophoresis (SDS–PAGE). Purified reBnCPI can efficiently inhibit the protease activity of papain and ficin toward BANA (*N*α-benzoyl-L-arginine-2-naphthyamide), as well as the mycelium growth of some important plant pathogenic fungi. The data further contribute to our understanding of the molecular functions of BnCPI.

## 1. Introduction

Cysteine protease inhibitors or cystatins of plant origin are called phytocystatins; they display multiple functions in seed formation and germination, plant growth and development, as well as plant resistance/tolerance to biotic/abiotic stresses [1,2,3]. For almost 30 years, phytocystatins and their roles in plant–pest interactions attracted extensive international concern and study. A deeper and more nuanced understanding of phytocystatins was created from the increasing amount of data and related research. Some phytocystatins can directly impair herbivore growth, as evidenced by impaired growth of these pests when reared on an artificial diet containing cystatins or transgenic plants with exogenous cystatin genes [4,5,6]. Cystatins of rice, maize and taro can increase plant resistance to various phytopathogenic nematodes [7,8,9]. Many phytocystatins can also directly inhibit the growth of phytopathogenic fungi [2]. Some phytocystatins (such as CeCPI, HvCPI-1) with antifungal activity also inhibit the growth of nematodes and herbivores [7,10,11,12]. Transgenic tobacco over-expressing potato sporamin and taro cystatin displayed increased resistance to both insects and phytopathogens, including *Helicoverpa armigera*, *Erwinia carotovora* and *Pythium aphanidermatum* [13]. These reports supply direct, substantial evidence for the potential of phytocystatins in improving plant resistance.

The inhibition mechanism of phytocystatins on different pests has also been explored. Cysteine protease (CP) is a major digestive enzyme in many herbivores and nematodes; it plays an important role in reproduction, development, tissue invasion, pathogenesis and immune invasion [14,15,16,17]. The harmful effect of phytocystatins on nematodes and herbivores was thought to inhibit the activity of CP of these animals. However, the mechanism of growth inhibition of phytocystatins on fungi is still unclear. Some researchers have proposed that it blocks indigenous proteinase activity of fungi [11,18], whereas others found that the inhibition is not associated with its cysteine proteinase inhibitory properties [10]. These discoveries indicate that the inhibition mechanisms of different phytocystatins toward fungi are different.

Ramie (*Boehmeria nivea* L.) is a perennial herbaceous plant of the family Urticaceae. It is also called “China grass” since it has been cultivated in China for over 6000 years [19]. Ramie is an important natural fiber crop planted mainly in China, India and other Southeast Asian and Pacific Rim countries [20]. Traditionally, ramie was planted solely for harvesting bast fibers. Recently, ramie leaves and shoots have also been used as fodder for beef cattle and geese because of the plant’s high crude protein content [21]. Root lesion disease (RLD), a destructive root disease that is caused by the nematode *Pratylenchus coffeae*, severely impairs the growth and yield of ramie [22]. The lack of knowledge on ramie RLD resistance mechanisms or genes severely hinders efforts in effectively breeding and utilizing nematode-resistant ramie. More recently, a ramie cystatin gene *BnCPI* (Cysteine protease inhibitor, Unigene11292) was found to be regulated in *P. coffeae*-infected resistant ramie but not in a susceptible cultivar, which suggested that *BnCPI* may be involved in pest resistance [23]. In order to decipher the biological function of *BnCPI*, it is very essential to clone and characterize the gene and active protein. This study reported the molecular cloning, sequence analysis, recombinant expression and biochemical and antifungal activity study of this gene and its corresponding protein.

## 2. Materials and Methods

### 2.1. Plant Growth and Sampling

Ramie varieties Qingdaye (QDY), Zhongzhu No.1 (ZZ1) and Heipidou (HPD), which were resistant, moderately susceptible, and susceptible to *P. coffeae*, respectively, were used in this study. Seedlings were prepared with the cutting propagation method [23]. Two weeks later, when the roots were about 20 cm length, roots of a single plant were sampled and immediately frozen in liquid nitrogen and stored at −80 °C.

### 2.2. DNA and RNA Extraction and cDNA Synthesis

Root tissues of ramie in a 2 mL Eppendorf tube (pre-chilled in liquid nitrogen) were ground to fine powder using a Tissuelyser-24 Multi-Sample Tissue Grinder (Shanghai Jingxing, Shanghai, China). Total DNA and RNA were extracted using a Plant DNA Mini Kit and an EASYspin Plus Total RNA Kit (both from Aidlab, Beijing, China), following the manufacturer’s protocol. A Nanodrop 2000 spectrophotometer (Thermo Fisher Scientific, Waltham, MA, USA) was used to measure DNA and RNA concentration, and first-strand cDNA samples were synthesized from about 1 μg of the total RNA using a RevertAid First Strand cDNA Synthesis Kit (Thermo Fisher Scientific).

### 2.3. Molecular Cloning of the BnCPI Gene

A 787-bp cDNA fragment of the *BnCPI* gene was obtained from transcriptome sequencing data of ramie [23]. The sequences contained an open reading frame (ORF)-encoded ramie homolog of the cysteine protease inhibitor. Based on this sequence, 3′-RACE (rapid amplification of cDNA ends) was performed to amplify the 3′-end of this gene using a SMART™ RACE cDNA Amplification Kit (Clontech, Mountain View, CA, USA) according to the manufacturer’s guidelines. Two internal gene-specific forward primers, cpi3-1 (5′-GAT GGC GGT GTC AAG AAG GTT TAC GA-3′) and cpi3-2 (5′-AAG GTC TGG GAA AAG TTG TGG TTG AA-3′), were assigned from the known cDNA fragment of *BnCPI*. Briefly, first-strand cDNA was synthesized with 3′-CDS primer A, and then amplified by PCR using cpi3-1 as a forward primer and UPM (Universal Primer A mix) as the reverse primer. Subsequently, the product of the first round of PCR was diluted 50 times, and used as a template in the second-round PCR amplification using cpi3-2 and UPM. The PCR products were separated on 1% agarose gels, purified, cloned into pMD-18 vector and sequenced by Shanghai Sangon Biotechnology Co. Ltd. (Shanghai, China). The resulting full-length cDNA of *BnCPI* was deposited in the NCBI (National Center for Biotechnology Information) GenBank with accession number KT438742.1.

### 2.4. Sequences Analysis and Phylogenetic Analysis

The online ORF finder program of NCBI (https://www.ncbi.nlm.nih.gov/orffinder/) was used to search the entire ORF sequences of *BnCPI*, while the online BLAST program of NCBI was used to search protein homology. Theoretical molecular weight (MW) and isoelectric point (pI) for BnCPI were predicted using the ExPASy tool (http://web.expasy.org/compute_pi/); signal peptide prediction was performed using SignalP 4.1 (http://www.cbs.dtu.dk/services/SignalP/); and secondary structure was predicted using SOPMA [24]. The conserved domain was searched against the NCBI Conserved Domain Database [25].

A set of primers (cpif: 5′-CGC AGA AAA GTA AAA GCA-3′ and cpir: 5′-TCC ACC AAA GAC GAA TGA-3′) was assigned for PCR amplification of ORF fragments in different ramie cultivars. Primers cpif and cpir were located upstream of the start codon and downstream of the stop codon of the ORF, respectively. PCR was performed in an ETC-811 PCR instrument (Eastwin, Beijing, China). The reaction mixture contained 25 μL of 2× Taq PCasterMix (Aidlab, Beijing, China), 4 μL of each primer (10 μmol/μL), 2 μL of genomic DNA or cDNA (10 μg/μL), and the total volume was adjusted to 50 μL with ddH_2_O. The PCR amplification procedure consisted of 94 °C for 4 min, 35 cycles of denaturation at 94 °C for 45 s, annealing at 50 °C for 1 min, and extension at 72 °C for 1 min, with final extension at 72 °C for 10 min. The PCR products were separated by electrophoresis on 1.5% agarose gels and visualized by staining with Gold View (Applygen, Beijing, China). Amplicons were sequenced by Tsingke Company (Tsingke, Beijing, China). *BnCPI* and phytocystatins from other plants downloaded from the NCBI website were aligned using MEGA 6.0 software [26]. The resulting alignment was trimmed and a phylogenetic tree was constructed using the neighbor-joining method with 100 bootstrapping. The deduced amino acid sequence of *BnCPI* was also aligned with those of other phytocystatins available in the EMBL-EBI database ClustalW2 (http://www.ebi.ac.uk/Tools/msa/muscle/), set at default parameters.

### 2.5. Expression of a Recombinant *BnCPI* in Escherichia coli

The ORF of *BnCPI* contains many rare codons (Figure S1a) that will impair expression efficiency in a recombinant expression. Gene codon optimization was performed by synthesizing sequences encoding amino acids identical to those encoded by *BnCPI* but without rare codons (Figure S1b). ORF gene synthesis was performed by the Tsingke Co. (Tsingke, Beijing, China). The optimized gene fragment was cloned into the expression vector pSmart-I (General Biosystems, USA), which carried 6× His-tag and SUMO to facilitate solution and purification of the recombinant expressed protein. The recombinant vector was subsequently transformed into *Escherichia coli* BL21 (DE3). Positive clones containing the recombinant vector were screened on ampicillin. Cells of positive clones were grown at 37 °C in LB liquid medium with 220 rpm until OD600 reached 0.6–0.8. Subsequently, IPTG (isopropyl β-d-1-thiogalactopyranoside) was added to a final concentration of 0.5 mM to induce expression of *BnCPI* at 37 °C for 4 h or at 15 °C for 16 h.

After culture, cells were collected by centrifugation, suspended in 25 mM Tris buffer (containing 300 mM NaCl, pH 8.0), and sonicated by an ultrasonic processor for 2 min. The fragmentized cell suspension was centrifuged at 12,000 rpm at 4 °C for 15 min. The resultant suspension and precipitate were separated with sodium dodecyl sulfate polyacrylamide gel electrophoresis (SDS–PAGE) to check the recombinant expressed BnCPI (reBnCPI). Then, recombinant protein was treated with SUMO protease to remove the SUMO protein and purified by a Ni–NTA affinity column (GE Healthcare, Russellville, AR, USA) using an ÄKTA-prime system (GE Healthcare).

### 2.6. Protease Inhibitory Activity Assay

Cysteine protease inhibitory activity of reBnCPI toward papain (EC 3.4.22.2), ficin (EC 3.4.22.3) and bromelain (EC.3.4.22.32) (all from Sigma-Aldrich, St. Louis, MO, USA) were tested using a method described previously [15]. The inhibitory activity of reBnCPI was recorded as residual enzyme activity in the presence of inhibitor. In the control treatment, equal volumes of the corresponding buffer were used instead of reBnCPI. There were three replicates in each treatment and the experiment was repeated twice.

### 2.7. Antifungal Activity Assay of reBnCPI

Two pathogens of the ramie plant, *Pythium vexans* causing brown root rot [27] and *Alternaria alternata* causing leaf spot [28], as well as two other important plant pathogens (*Fusarium oxysporum* and *Botrytis cinerea*, isolates of which are kept in our laboratory), were used for the growth inhibition assay. The in vitro growth inhibition assays were performed as described by Pernas et al. [29]. Fungal strains were grown on potato dextrose agar (PDA) medium for 3–7 days. For *F. oxysporum*, *A. alternata* and *B. cinerea*, spores were collected with 1/3 PDB (PDA medium without agar) and diluted to a concentration of 10^4^ spores/mL. For *P. vexans*, hyphae were collected with 1/3 PDB in a 2-mL Eppendorf tube, homogenized and diluted to a concentration of 10^4^ CFU/mL. The reBnCPI (after filtration sterilization) was added to the suspension to produce a final concentration of 20–80 μg/mL. Fungicides carbendazim, jinggangmycin, fluazinam and mefenoxam were added to the suspensions of *F. oxysporum*, *A. alternata*, *B. cinerea* and *P. vexans* (final concentration 50 μg/mL), respectively, to serve as positive controls. Fungal suspensions without fungicides and reBnCPI were used as negative controls, and buffer (25 mM Tris, 300 mM NaCl, pH 8.0) used to dissolve reBnCPI was added instead of reBnCPI or fungicides. Then, spores or hyphae suspension of 200 μL were cultivated on a sterile 96-well microtiter plate at 25 ± 1 °C for 48 h. Fungal growth was then monitored by measuring absorbance at 492 nm (Infinit200 Pro, Tecan, Männedorf, Switzerland) and checked under a Nikon AZ100 microscope (Nikon Co., Tokyo, Japan). Results were expressed as the percentage of relative growth in the absence of the reBnCPI. Each treatment was replicated three times, and the experiment was repeated twice.

## 3. Results

### 3.1. Sequence Analysis

**Figure 1:**
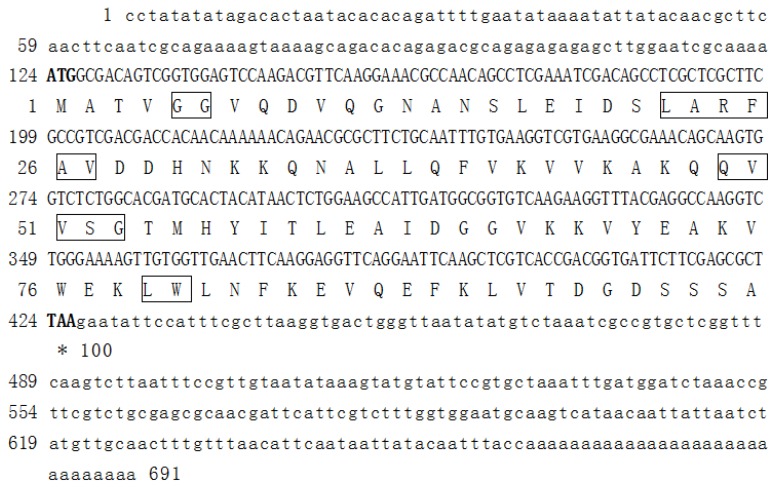
Nucleotide and deduced amino acid sequences of the identified full-length cDNA of BnCPI. Nucleotide in bold text indicates the start and stop codons. Several motifs of phytocystatins were detected in this sequence, such as GG, LARFAV, QVVSG and LW. The respective motifs are boxed.

The cDNA sequence of the *BnCPI* gene was 691 bp in length, including 123 bp of 5′ untranslated region (UTR), 303 bp of ORF and 265 bp of the 3′ UTR. The ORF likely encoded a deduced 100-residue amino acid (AA) sequence (Figure 1). The mature protein had a molecular weight of 11.06 kDa and pI of 6.0, as predicted by the ExPASy tool. No signal peptide was found when the AA sequence was analyzed with SignalP software. A conserved-domain database (CDD) search showed that the deduced BnCPI protein sequence was identical to the conserved cystatin-like domain (CY domain: cd00042). BnCPI contained most of the highly conserved blocks that are essential for cysteine proteinase activity, including the GG (Gly^5^-Gly^6^), the reactive site motif QxVxG (Q^49^V^50^V^51^S^52^G^53^), the W residue (W^80^) and the highly conserved [LVI]-[AGT]-[RKE]- [FY]-[AS]-[VI]-x-[EDQV]-[HYFQ]-N block (L^22^G^23^R^24^ F^25^A^26^V^27^ D^28^D^29^H^30^ N^31^) (Figure 1). The secondary structure of the coded protein mainly consisted of α-helices and random coils (Figure S2a). The tertiary structure prediction of the coded protein indicated that the space structure mainly contained α-helices, β-turns and β-sheets (Figure S2b).

**Figure 2:**
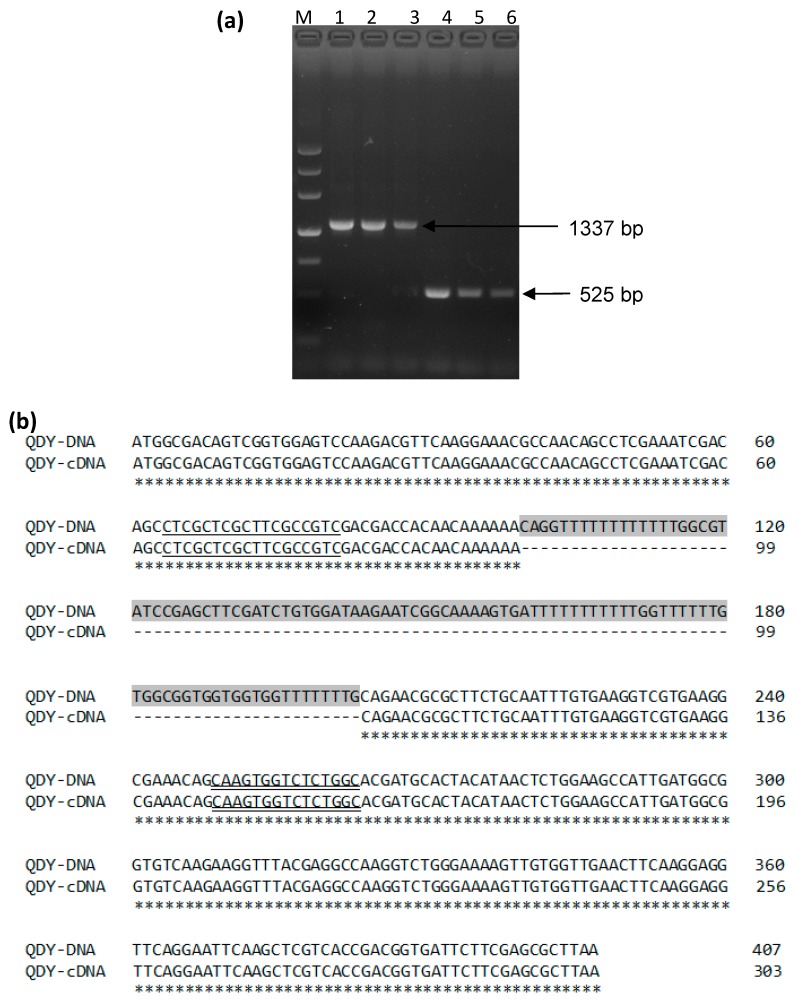
(**a**) Electrophoresis of PCR amplification fragments of BnCPI from cDNA and genome DNA on 1.5% agarose gel. M, DNA marker III; 1‒3, fragments amplified from DNA of ZZ1, HPD and QDY respectively; 4‒6, fragments amplified from cDNA of ZZ1, HPD and QDY, respectively; (**b**) Alignment of genome DNA (accession No. MF153097) and cDNA (accession No. KT438742) sequences of BnCPI from ramie (cultivar. QDY). The shaded nucleic acids indicate an intron of 104 bp in length. The sequences encoding the LARFAV motif and the reactive site QxVxG are underlined and double underlined, respectively.

**Figure 3:**
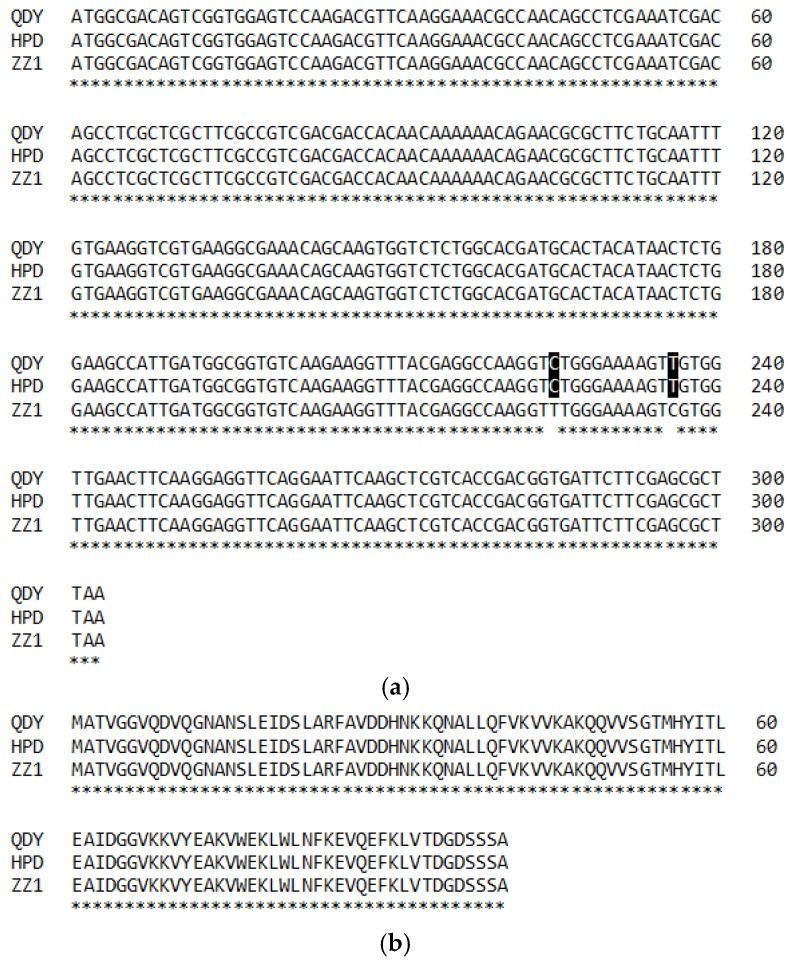
(**a**) Alignment of cDNA and (**b**) deduced amino acid of BnCPI from three ramie cultivars, QDY, HPD and ZZ1. Two nucleic acids (positions 225 and 236) in ZZ1 differ from QDY and HPD (nucleic acid shaded). The deduced amino acids of three cultivars are identical.

Both the genome DNA and the cDNA sequences of *BnCPI* were amplified from different ramie cultivars using the primers cpif and cpir (Figure 2a). Alignment of cDNA and the genome DNA sequences indicated the presence of a 104 bp intron between the sequence encoding the LARFAV motif and the reactive site QxVxG (Figure 2b). The ORF sequences of *BnCPI* from the cultivars QDY and HPD were found to be identical, whereas two nucleic acids (positions 225 and 236) were identified in cultivar ZZ1 that differed from those in QDY and HPD (Figure 3a), while the encoded amino acids were identical (Figure 3b).

### 3.2. Homology Analysis of BnCPI

Analysis of the BLAST results (11 May 2017) revealed that the deduced amino acid sequence of BnCPI shared the highest identity (76%) with sequences of cystatin from *Glycine max* (accession nos. XP_003543365 and AAA97905) and *Ricinus communis* (accession No. XP_002523225), followed by sequences of cystatin from *Glycine soja* (accession no KHN35444), *G. max* (accession No. NP_001239817) and *Hevea brasiliensis* (accession No. ACZ02398), all of which shared 75% identity with *BnCPI*. These were followed by sequences of cystatin from *Vigna unguiculata* (accession No. CAA79954) and *Cajanus cajan* (accession No. XP_020236689), both of which shared 74% identity with *BnCPI*.

**Figure 4:**
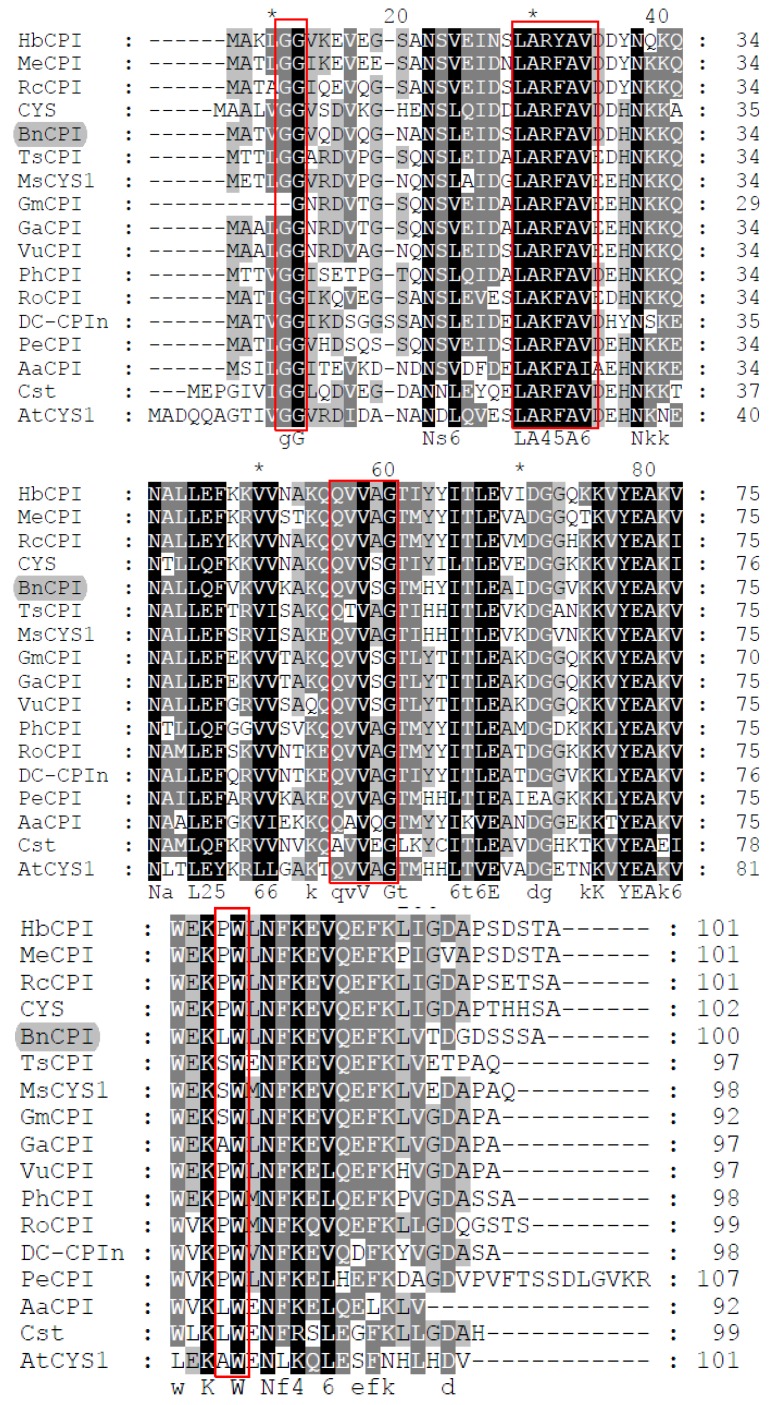
Alignment of the deduced amino acid sequence of *BnCPI* (ALG38347) with the sequences of phytocystatins (with molecular mass of about 12 kDa) from other plants; they are *Hevea brasiliensis* HbCPI (ACZ02398), *Manihot esculenta* MeCPI (AAF72202), *Pelargonium hortorum* PhCPI (ABG81097), *Trifolium subterraneum* TsCPI (GAU21037), *Rumex obtusifolius* RoCPI (CAD21441), *Medicago sativa* MsCYS1 (AAZ98791), *Castanea sativa* CYS (CAA11899), *Populuuphratica* PeCPI (AGX28139), *Glycine max* GmCPI (KHN35444), *Glycine soja* GaCPI (KHN35444), *Vigna unguiculata* VuCPI (CAA79954), *Ricinus communis* RcCPI (XP_002523225), *Dianthus caryophyllus* DC-CPIn (AAK30004), *Arabidopsis thaliana* AtCYS1 (CAA03929), *Carica papaya* Cst (CAA50437) and *Ambrosia artemisiifolia* AaCPI (AAA32672). The identical and partial-conservation residues are shaded in black and gray, respectively. Red boxes show conserved motifs of phytocystatins. The alignment was performed by ClustalW program and was shaded using the Gene Doc software.

As shown in Figure 4, alignment of *BnCPI* with sequences of phytocystatins from other plants confirmed that BnCPI and *R. communis* cystatin were most similar. BnCPI contained most of the typical features of other phytocystatins, such as the GG doublet and LARFAV motif in the N-terminal region, and the central signature motif QxVxG (also as the active site), but it lacked the PW motif contained in some of the other cystatins from plants and animals. Here, it was revealed by multiple alignments that the PW motif was replaced by the LW motif in the BnCPI amino acid sequences, which is identical to the phytocystatin of papaya (accession No. CAA50437) and ragweed (accession No. AAA32672).

### 3.3. Recombinant Expression of BnCPI

**Figure 5:**
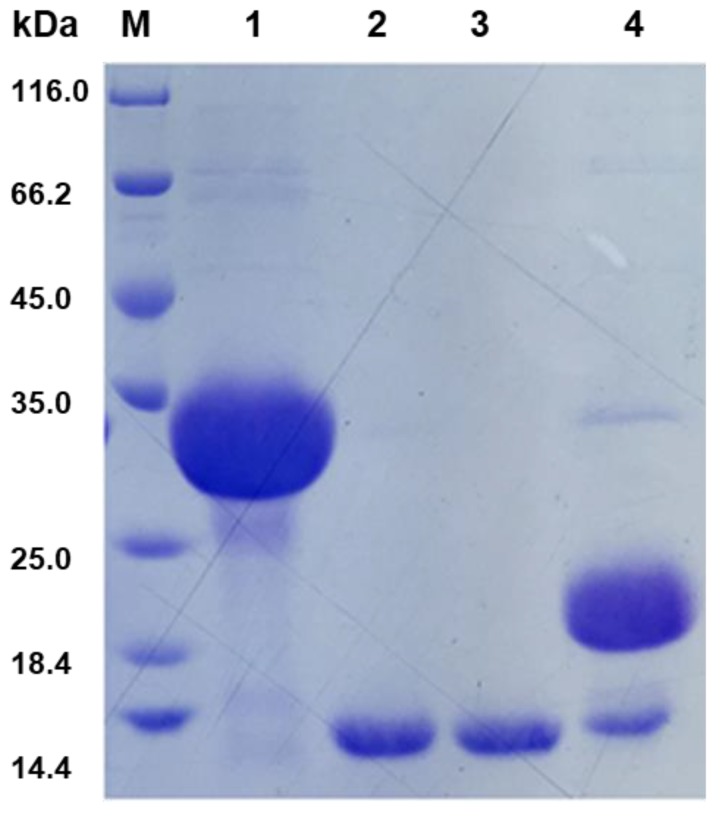
Analysis of recombinant expressed BnCPI (reBnCPI) on 12% sodium dodecyl sulfate polyacrylamide gel electrophoresis (SDS–PAGE). M: protein marker; 1: reBnCPI with SUMO and His tag (before enzyme digestion); 2 and 3: reBnCPI without SUMO and His tag, respectively (after enzyme digestion); 4: SUMO and His tag (after enzyme digestion).

The ORF sequence was optimized and subsequently introduced into the pSmart-I vector, then expressed in *E. coli* BL21 (DE3). Overexpression of *BnCPI* was induced by adding IPTG (final concentration 0.5 mM) to the medium. Expressed recombinant BnCPI was not secreted to the liquid medium. Bacterial cells were collected by centrifugation, homogenized by ultrasonic crash and purified by affinity chromatography. After removing SUMO and further purification, the resultant protein was analyzed using SDS–PAGE electrophoresis. A single protein band with a molecular mass of about 11.4 kDa was obtained (Figure 5), which was slightly higher than the deduced protein size (11.06 kDa) for BnCPI (described above). The results indicated that successful prokaryotic expression of *BnCPI* was achieved.

A phylogenetic tree (Figure S3) constructed with the amino acid (AA) sequences of BnCPI and 51 other cystatins from different plant species revealed that the BnCPI sequences are most closely related to cystatin from *V. unguiculata*, followed by cystatin from *Vigna radiata*, *G. max* and *G. soja*. These results are in agreement with those generated by the BLAST research analysis.

### 3.4. Protease Inhibitory Activity of reBnCPI

**Figure 6:**
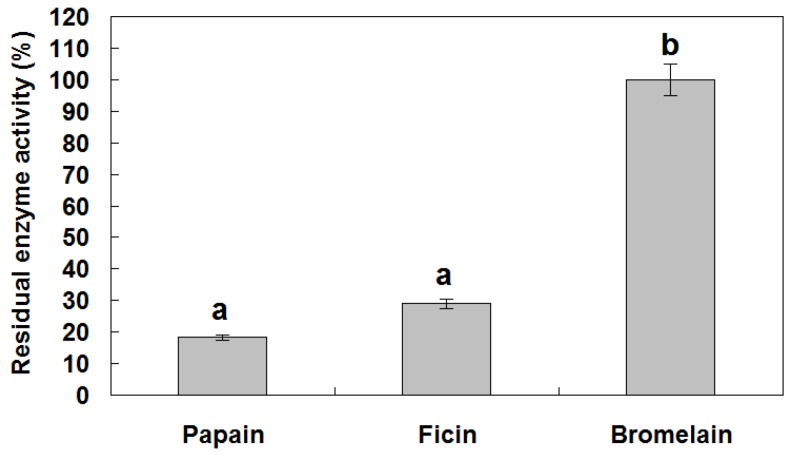
Assay of inhibition activity of purified reBnCPI toward papain, ficin and bromelain. The inhibition was expressed as residual enzyme activity in the presence of BnCPI at 100 μg/mL, and *N*α-benzoyl-dl-arginine β-naphthylamide (BANA) was used as substrate. Vertical bars represent standard deviations. Different small letters on bars represent significant difference at 5% level (Duncan’s multiple range test).

To determine whether the recombinant BnCPI inhibits cysteine proteinase, the purified reBnCPI (≈10 μg) was pre-incubated with papain, ficin and bromelain, and then the residual enzyme activities on *N*α-benzoyl-DL-arginine β-naphthylamide (BANA) were tested as above. For papain and ficin, only about 18.2% and 29.0% enzyme activities remained after treatment, respectively, whereas for bromelain, reBnCPI showed no obvious inhibition activity (Figure 6). These data indicate that purified reBnCPI has strong inhibitory effects on enzymes with cysteine proteinase activity, and that functional expression was achieved.

### 3.5. Antifungal Activity of reBnCPI

**Figure 7:**
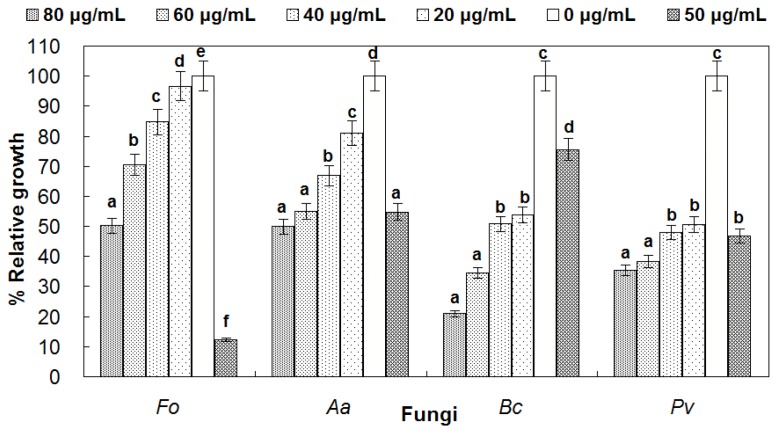
Growth inhibition assays of four fungal pathogens by reBnCPI. Fo, *Fusarium oxysporum*; Aa, *Alternaria alternata*; Bc, *Botytis cinerea*; and Pv, *Pythium vexans*. reBnCPI was added to the fungal suspension to a final concentration of 0, 20, 40, 60 or 80 μg/mL. Fungicides carbendazim, jinggangmycin, fluazinam and mefenoxam were added to the suspension of Fo, Aa, Bc and Pv (final concentration 50 μg/mL), respectively, to serve as positive control. Vertical bars represent standard deviations. Different small letters on bars represent significant difference at 5% level (Duncan's multiple range test).

Antifungal activity assays were performed with different concentrations of purified reBnCPI on four plant pathogenic fungi (*F. oxysporum*, *A. aternata*, *B. cinerea* and *P. vexans*) cultures. The results indicated that reBnCPI inhibited the growth of all four phytopathogens, but the inhibitory effect varied depending on the fungal species (Figure 7). Among these four fungi, *B. cinerea* was most sensitive to reBnCPI. Concentrations of reBnCPI of 20 μg/mL (1.81 μM) slowed the growth rate of *B. cinerea* to 54% of that exhibited by *B. cinerea* cultivated in medium without reBnCPI; at concentrations of 80 μg/mL (7.23 μM), the *B. cinerea* growth rate was only 21% of that cultivated in medium without reBnCPI. Growth rates of *F. oxysporum* and *A. alternata* were reduced by about 50% at a concentration of reBnCPI of 80 μg/mL; that is, the 50% growth inhibition (EC_50_) for these two pathogens was 80 μg/mL. Moreover, the inhibition efficiency of reBnCPI (at 80 μg/mL) was slightly higher than that of jinggangmycin (50 μg/mL). In the case of the root rot pathogen *P. vexans*, growth rates fell by 50% at reBnCPI concentrations of 20 μg/mL and 35% at concentrations of 80 μg/mL, indicating that *P. vexans* is not very sensitive to BnCPI. For all four fungi, however, the growth rate declined as the concentrations of reBnCPI increased.

The inhibitory effects that reBnCPI exhibited on the growth of these four phytopathogenic fungi were also checked under microscopy (Figure S4). In the negative control (without reBnCPI) treatments, all four fungal species grew fast, with more mycelia than that present with BnCPI and fungicides. Cultured in the broth with reBnCPI, *A. alternata* and *P. vexans* exhibited reduced mycelia, whereas *F. oxysporum* and *B. cinerea* exhibited abnormal hyphal growth besides reduced development (Figure S4b,e,h,k). reBnCPI did not suppress spore germination of *A. alternata*, *F. oxysporum* and *B. cinerea* at the tested concentrations.

## 4. Discussion

In this study, we have cloned the full-length cDNA encoding a cystatin gene (*BnCPI*) of ramie for the first time. We also compared *BnCPI* sequences of three ramie cultivars with different nematode resistance. Interestingly, *BnCPI* genes of different ramie cultivars with different nematode resistance have identical AA sequences, although there were very few nucleotide differences in the cDNA sequence. Supposing BnCPI plays an important role in the ramie–nematode interaction, the resistance difference of cultivars may be due to different transcriptional regulation or expression regulation of this protein. For instance, expression of two cystatins (WC4 and WC5) in resistant and susceptible wheat cultivars were differently induced during *Tilletia indica* infection; and the different expressions were regulated by Jasmonic acid (JA) signal [30].

An accurate classification of BnCPI will help readers to understand it comprehensively. Based on the molecular mass (11.4 kDa) and other molecular characteristics of BnCPI (described above), we concluded that it should be clustered into group I. Subgroups (Group I, Group II and Group III) of the phytocystatin family were generated on the basis of phylogenetic analysis [31]. Introns are common in the coding regions of cystatins from plants and animals, and some phytocystatins contain more than one intron [32,33,34,35]. Intron–exon structure is also often used to characterize cystatin genes; those lacking introns in their ORF are categorized as MCOG C, whereas those containing three introns are classed into subgroup B1, and those with only one intron in the DNA sequence—located between the coding sequences for the conserved motifs LARFAV and QxVxG—are placed in either MCOG A or subgroup B2 [36]. According to this principle, BnCPI belongs to subgroup MCOG A.

The recombinant BnCPI exhibited inhibition activity toward both papain and ficin, but not bromelain. Plant cystatins are generally poor inhibitors of bromelain [37,38], but AcCYS1 (protein ID ACL14375) from pineapple showed strong inhibition activity toward bromelain from the pineapple stem and fruit due to the presence of “an extended N-terminal trunk (AE-rich NTT) of 63 residues rich in alanine and glutamate” [39]. A previously identified pineapple stem cystatin (protein ID AAQ07259) lacking an AE-rich NTT exhibited no inhibitory effects on bromelain [40]. The cysteine proteinase inhibition activity of BnCPI is in accordance with that of most of the phytocystatins.

Phytocystatins are apparently reversible, competitive inhibitors of papain [36]. However, there are some phytocystatins, such as HbCPI, FaCPI-1, Zm, OC-I, OC-II and CaCPI that are non-competitive inhibitors of papain [36,41]. The inhibition pattern of reBnCPI to papain was not characterized in this study. Since BnCPI has higher similarity to cystatins from soybean (L1, protein ID AAA97905) and rubber tree (HbCPI, protein ID ACZ02398), it is also probably a competitive inhibitor of papain.

Recombinant BnCPI exhibited varied growth inhibition on *F.oxysporum*, *B. cinerea*, *A. alternata* and *P. vexans*. Differential inhibitory effect of a phytocystatin on different fungi is common for phytocystatins. Cystatins from chestnut (CSC), wheat (TaMDC1), taro (CeCPI), sugarcane (canecystatin), barley (HvCPI‒1 to HvCPI‒13), strawberry (FaCPI-1), kiwifruit (KCPI1 or rCPI), amaranth (AhCPI), cacao (TcCys), Siam tulip (CaCPI) and sesame (SiCYS) have all been shown to have toxic effects on mycelium growth of various species of fungi [2]. For Hv-CPI1, FaCPI-1 and KCPI, 50% growth inhibition of *B. cinerea* occurred at concentrations of 1.5 μM, 1.9 μM and 2.7 μM, respectively [10,42,43], which are close to that of BnCPI (~1.81 μM). For *F. oxysporum*, 50% growth inhibition of HvCPI-1, AhCPI and CaCPI occurred at concentrations of 2.14 μM, 2.28 μM and 13 μM, respectively [10,18,44]. The 50% growth inhibition of reBnCPI on *F. oxysporum* was about 7.23 μM, which is higher than that reported for other phytocystatins. To our knowledge, the maximum EC_50_ reported for phytocystatins on *F. oxysporum* are those of HvCPI-9 (5.94 μM) and HvCPI-11 (6.0 μM) [45]. The toxicity of phytocystatins on *A. alternata* and *P. vexans* has not been studied; however, KCPI1 inhibits the growth of *A. radicina* almost completely at a concentration of 23.1 μM, and displays 50% growth inhibition at a concentration of 2.2 μM [43], and CeCPI significantly inhibits mycelia growth of *P. aphanidermatum* at concentrations of 150 μg/mL (~5.2 μM) to 200 μg/mL (≈6.9 μM) [11]. These data indicate that phytocystatins may also be capable of inhibiting mycelial growth of *Pythium* spp. and *Alternaria* spp. However, the mechanisms responsible for the growth inhibition activity of reBnCPI on different fungi require further, detailed elucidation.

## 5. Conclusions

In summary, *BnCPI*, a gene encoding a phytocystatin in ramie, was cloned successfully with the RACE technique. The full-length cDNA of *BnCPI* (accession No. KT438742) contained an ORF of 303 bp, encoding a protein of 100 amino acids with deduced molecular mass of 11.06 kDa and a theoretical pI of 6.0. The corresponding genome DNA consisted of an intron of 104 bp in the coding region. The ORF of *BnCPI* was subcloned into an expression vector and was expressed successfully in *E. coli*. The recombinant expressed protein, reBnCPI, exhibited strong cysteine proteinase inhibition activity on papain and ficin, as well as on the growth of some phytopathogenic fungi. These results further contribute to our understanding of the molecular functions of BnCPI in ramie growth and development, and resistance to biotic and abiotic stresses.

## Supplementary Materials

The following are available online at www.mdpi.com/2073-4425/8/10/265/s1, Figure S1: rare codon analysis (A) and optimization (B) of BnCPI for recombinant expression; Figure S2: BnCPI secondary and tertiary structure predicted by SOPMA and SWISS-MODEL software, respectively; Figure S3: uprooted phylogenetic tree of cystatins from ramie (BnCPI) and other plants constructed by MEGA 6.0 using the neighbor-joining method (NJ) with 1000 bootstrap replicates; Figure S4: microscopic photographs of four phytopathogen’s growth suppression by recombinant BnCPI (reBnCPI) and fungicides.
