# Supplementary material for: Molecular Cloning, Recombinant Expression and Antifungal Activity of BnCPI, a Cystatin in Ramie (Boehmeria nivea L.)
Source: Genes (Basel). 2017 Oct 11;8(10):265. doi: 10.3390/genes8100265 (PMC5664115; doi:10.3390/genes8100265)
Supplement: Supplementary file 1 [file genes-08-00265-s001.zip › Supplementary File(s)/Figure S1.doc]

Figure S1

**(a)**

**(b)**

Figure S1: Rare codon analysis (A) and optimization (B) of *BnCPI* for recombinant expression.
