# Supplementary material for: Molecular Cloning, Recombinant Expression and Antifungal Activity of BnCPI, a Cystatin in Ramie (Boehmeria nivea L.)
Source: Genes (Basel). 2017 Oct 11;8(10):265. doi: 10.3390/genes8100265 (PMC5664115; doi:10.3390/genes8100265)
Supplement: Supplementary file 1 [file genes-08-00265-s001.zip › Supplementary File(s)/Figure S2.doc]

Figure S2

**(a)**

**(b)**

Blue: α-helix; Red: Extended strand; Green: β-turn; Orange: Random coil.

Figure S2. BnCPI secondary and tertiary structure predicted by SOPMA and SWISS-MODEL software, respectively. A: 46% amino acid (aa) formed α-helix; 21% aa were for Extended strand; 10% aa formed β-turn and 23% aa formed Random coil. B: The three-dimensional structure mainly contained α-helices, β-turns and β-sheets.
