# Supplementary material for: Molecular Cloning, Recombinant Expression and Antifungal Activity of BnCPI, a Cystatin in Ramie (Boehmeria nivea L.)
Source: Genes (Basel). 2017 Oct 11;8(10):265. doi: 10.3390/genes8100265 (PMC5664115; doi:10.3390/genes8100265)
Supplement: Supplementary file 1 [file genes-08-00265-s001.zip › Supplementary File(s)/Figure S3.doc]

AAA97905 *Glycine max*

KHN35444 *Glycine soja*

AAL56612 *Vigna radiata*

**ALG38347 *Boehmeria nivea***

AAQ14319 *Vigna unguiculata*

CAA11899 *Castanea sativa*

AAF72202 *Manihot esculenta*

ACZ02398 *Hevea brasiliensis*

ADB02894 *Jatropha curcas*

XP 002523225 *Ricinus communis*

AAZ98791 *Medicago sativa*

CBX19819 *Arachis hypogaea*

CAN78115 *Vitis vinifera*

AAD33907 *Artemisia vulgaris*

AAA32672 *Ambrosia artemisiifolia*

AEK84226 *Cucurbita maxima*

CAA50437 *Carica papaya*

AAS21216 *Aegiceras corniculatum*

BAH47556 *Spinacia oleracea*

AAK30004 *Dianthus caryophyllus*

CAD21441 *Rumex obtusifolius*

ADD69946 *Knorringia sibirica*

AEQ54767 *Coffea canephora*

AAC37479 *Brassica campestris*

ABN42208 *Cakile maritima*

CAH57539 *Populus tremula*

AAY41807 *Populus tomentosa*

ACO39782 *Populus balsamifera*

CAA89697 *Ricinus communis*

AAO19652 *Malus domestica*

ABG89856 *Amaranthus hypochondriacus*

AGF33489 *Curcuma alismatifolia*

ACF06548 *Elaeis guineensis*

AAL79831 *Sandersonia aurantiaca*

Q4GZT8 *Fragaria ananassa*

AKB91191 *Theobroma cacao*

AAK15090 *Sesamum indicum*

AAD13812 *Ipomoea batatas*

AAF23126 *Solanum lycopersicum*

AAU81597 *Petunia x hybrida*

AAQ07259 *Ananas comosus*

ACY30464 *Ficus pumila* var. *awkeotsang*

Q945Q1 *Arabidopsis thaliana*

AAB24010 *oryzacystatin*

CAJ20024 *Zea mays*

BAB18768 *Triticum aestivum*

AAM78598 *Saccharum officinarum*

CAA72790 *Hordeum vulgare* sub.sp. *vulgare*

AAB71505 *Pyrus communis*

AAM88397 *Colocasia esculenta*

AHZ92268 *Malus prunifolia*

JE0308 *Helianthus annuus*

0.1

Figure S3

Uprooted phylogenetic tree of cystatins from ramie (BnCPI) and other plants constructed by MEGA 6.0 using the neighbor-joining method (NJ) with 1,000 bootstrap replicates.
