# Supplementary material for: Molecular Cloning, Recombinant Expression and Antifungal Activity of BnCPI, a Cystatin in Ramie (Boehmeria nivea L.)
Source: Genes (Basel). 2017 Oct 11;8(10):265. doi: 10.3390/genes8100265 (PMC5664115; doi:10.3390/genes8100265)
Supplement: Supplementary file 1 [file genes-08-00265-s001.zip › Supplementary File(s)/Figure S4.doc]

**Negative control	80 μg/mL reBnCPI	Positive control**

**(a)**

**(b)**

**(c)**

***Fo***

**(d)**

**(e)**

**(f)**

***Aa***

**(g)**

**(h)**

**(i)**

***Bc***

**(j)**

**(k)**

**(l)**

***Pv***

**Figure S4.** Microscopic photographs of four phytopathogens growth suppression by recombinant BnCPI (reBnCPI) at and fungicides. Fo, *Fusarium oxysporum*, Aa, *Alternaria alternata*, Bc, *Botytis cinerea*, and Pv, *Pythium vexans*. (a, d,g, and j) fungi growth without reBnCPI or Fungicides, (b, e, h, and k) with 80 μg /mL reBnCPI, and (c, f, i, and l) with 50 μg/mL fungicides. Fungicides carbendazim, jinggangmycin, fluazinam and mefenoxam were added to the suspension of *Fo*, *Aa*, *Bc*, and *Pv*, respectively, to serve as positive control.
